# Adverse consequences of immediate thrombolysis-related complications: a multi-centre registry-based cohort study of acute stroke

**DOI:** 10.1007/s11239-021-02523-2

**Published:** 2021-07-13

**Authors:** Thang S. Han, Giosue Gulli, Christopher H. Fry, Brendan Affley, Jonathan Robin, David Fluck, Puneet Kakar, Pankaj Sharma

**Affiliations:** 1 Institute of Cardiovascular Research, Royal Holloway University of London, Egham, TW20 0EX UK; 2 Department of Stroke, Ashford and St Peter’s NHS Foundation Trust, Chertsey, GU9 0PZ UK; 3 School of Physiology, Pharmacology and Neuroscience, University of Bristol, Bristol, BS8 1TD UK; 4 Department of Acute Medicine, Ashford and St Peter’s NHS Foundation Trust, Chertsey, GU9 0PZ UK; 5 Department of Cardiology, Ashford and St Peter’s NHS Foundation Trust, Chertsey, GU9 0PZ UK; 6 Department of Stroke, Epsom and St Helier University Hospitals, Epsom, KT18 7EG UK; 7 Department of Clinical Neuroscience, Imperial College Healthcare NHS Trust, London, W6 8RF UK

**Keywords:** Acute ischaemic stroke, Disability, Symptomatic intracranial haemorrhage, Mortality, Nosocomial infections

## Abstract

Complications following thrombolysis for stroke are well documented, and mostly concentrated on haemorrhage. However, the consequences of patients who experience any immediate thrombolysis-related complications (TRC) compared to patients without immediate TRC have not been examined. Prospectively collected data from the Sentinel Stroke National Audit Programme were analysed. Thrombolysis was performed in 451 patients (52.1% men; 75.3 years ± 13.2) admitted with acute ischaemic stroke (AIS) in four UK centres between 2014 and 2016. Adverse consequences following immediate TRC were assessed using logistic regression, adjusted for age, sex and co-morbidities. Twenty-nine patients (6.4%) acquired immediate TRC. Compared to patients without, individuals with immediate TRC had greater adjusted risks of: moderately-severe or severe stroke (National Institutes of Health for Stroke Scale score ≥ 16) at 24-h (5.7% vs 24.7%, OR 3.9, 95% CI 1.4–11.1); worst level of consciousness (LOC) in the first 7 days (score ≥ 1; 25.0 vs 60.7, OR 4.6, 95% CI 2.1–10.2); urinary tract infection or pneumonia within 7-days of admission (13.5% vs 39.3%, OR 3.2, 95% CI 1.3–7.7); length of stay (LOS) on hyperacute stroke unit (HASU) ≥ 2 weeks (34.7% vs 66.7%, OR 5.2, 95% CI 1.5–18.4); mortality (13.0% vs 41.4%, OR 3.7, 95% CI 1.6–8.4); moderately-severe or severe disability (modified Rankin Scale  score ≥ 4) at discharge (26.8% vs 65.5%, OR 4.7, 95% CI 2.1–10.9); palliative care by discharge date (5.1% vs 24.1%, OR 5.1, 95% CI 1.7–15.7). The median LOS on the HASU was longer (7 days vs 30 days, Kruskal–Wallis test: χ^2^ = 8.9, *p* = 0.003) while stroke severity did not improve (NIHSS score at 24-h post-thrombolysis minus NIHSS score at arrival = − 4 vs 0, χ^2^ = 24.3, *p* < 0.001). In conclusion, the risk of nosocomial infections, worsening of stroke severity, longer HASU stay, disability and death is increased following immediate TRC. The management of patients following immediate TRC is more complex than previously thought and such complexity needs to be considered when planning an increased thrombolysis service.

## Highlights

- In patients undergoing thrombolysis, compared with those without complications, patient with thrombolysis-related complications had 4 fold increase in early adverse outcomes.
- Stayed longer on hyperacute stroke units and mortality.
- They also had 5-fold increase risk of disability and requiring palliation at discharge.

## Introduction

Intravenous recombinant tissue plasminogen activator (rtPA), has been the standard thrombolysis treatment of acute ischaemic stroke (AIS) since the early 2000s in Europe [1, 2] and a few years earlier in the US [3]. The first decade into the treatment saw an increasing trend in the number of patients receiving thrombolysis [4, 5], subsequently followed by addition of this treatment to all eligible patients over 80 years of age [6, 7]. In recent years, the rate of thrombolysis administration has remained static in most countries where the proportion of all patients with stroke receiving thrombolysis (11–12%) has changed little between 2013 and 2020, well below the long-term target of 20% set by National Health Service (NHS) England [8]. Although outcomes have improved over the years [9, 10], many thrombolysis-related complications (TRC) remain a major issue, ranging from brain haemorrhage in the immediate stage to nosocomial infections, cardiac complications, seizures and deep vein thrombosis in later stages of treatment [11, 12]. The most feared immediate TRC is symptomatic intracranial haemorrhage (ICH), occurring in 7.7% of those treated with an rtPA compared to 1.8% in controls [11]. The Sentinel Stroke National Audit Programme (SSNAP) data of stroke patients in England and Wales published in 2013 reported symptomatic ICH occurred in 4.3% of patients < 81 years and 5.1% ≥ 81 years, followed by orolingual angioedema (< 2%) and extra-cranial bleed (< 1%) after thrombolysis [13].

Despite intensive research on the safety of rtPA, little is known about adverse consequences among patients who acquired immediate TRC. Most studies have investigated with the immediate outcome of, and from, bleeding following thrombolysis. In this study, we examined the association of immediate (and any) TRC with the risk of adverse outcome including: severity of stroke; ICH; nosocomial infections; worst level of consciousness (LOC) in the first 7-days following initial admission for stroke; length-of-stay (LOS) on hyperacute stroke units (HASUs); in-patient mortality and disability at discharge; as well as the level of support on discharge, including help with activities of daily living, new care home arrangement and palliation.

## Methods

### Study design, participants and setting

We performed analysis of prospectively collected data from the UK national register of stroke care (SSNAP). The data comprised clinical characteristics and care quality determinants of patients admitted to acute care hospitals in England and Wales [14]. Data from the present study were gathered from 3309 patients (2758 with AIS) admitted to four major UK hyperacute stroke centres in South East England between January 2014 and February 2016 [15, 16]. The sites included Ashford and St Peter’s (n = 1038), Frimley Park (n = 1010), Royal Surrey County (n = 612) and Epsom General (n = 649) hospitals. Outcome data from 451 (16.4% of AIS) consecutive patients undergoing thrombolysis were analysed in the present study.

SSNAP has approval from the Confidentiality Advisory Group of the Health Research Authority to collect patient data under section 251 of the NHS Act 2006 (no additional ethical approval was required).

### Socio-demographic factors and medical history

Demographic data were collected and documented by stroke consultants and nurse specialists; including age at arrival, gender and coexisting morbidities: atrial fibrillation (AF), hypertension, congestive heart failure, diabetes mellitus and previous stroke [14–16].

### Stroke diagnosis and severity

Stroke was diagnosed based on clinical presentation and brain imaging [14–16]. The severity of stroke symptoms at arrival was assessed by the National Institutes of Health for Stroke Scale (NIHSS) with a score range from no symptoms to severe stroke symptoms (NIHSS score = 0 to 42) [17].

### Thrombolysis and immediate TRC

Thrombolysis using the rtPA agent alteplase was performed in patients who fulfilled criteria for therapy including confirmed diagnosis of AIS, time from onset and without contra-indications [14–16]. TRC such as severe hypertension, acute orolingual angioedema, anaphylaxis and hyperacute haemorrhage were defined clinically. Identification of symptomatic ICH was based on imaging evidence of intracerebral haemorrhage in conjunction with a significant decline in neurological function [13, 14].

### Adverse consequences after immediate TRC

*Nosocomial infections* including urinary tract infection (UTI) and pneumonia acquired in hospital within 7-days of admission were recorded. *The worst LOC scores in the first 7-days following initial admission for stroke* were graded as: 0 = Alert keenly responsive, 1 = Not alert but arousable by minor stimulation, 2 = Not alert but require repeated stimulation to attend, and 3 = Respond only with reflex motor or autonomic effects/totally unresponsive [14]. The length of stay on HASUs as well as in-patient mortality were also documented. *Changes in severity* of *stroke* after thrombolysis were calculated as the difference between NIHSS score at 24-h minus NIHSS score on arrival.

*Disability at discharge* was evaluated using modified Rankin Scale (mRS) scores: 0 = no symptoms at all; 1 = no significant disability despite symptoms, able to carry out all usual duties and activities; 2 = slight disability, unable to carry out all previous activities but able to look after their own affairs without assistance; 3 = moderate disability; requiring some help, but able to walk without assistance; 4 = moderately severe disability, unable to walk without assistance and unable to attend to own bodily needs without assistance; 5 = severe disability, bedridden, incontinent and requiring constant nursing care and attention [18, 19].

### Level of care support planned at discharge

Details of the planned level of care support were recorded including: help for activities for daily living, the frequency of home visits, and joint care-planning between health and social care for post-discharge management. Information on decision to introduce palliative care by discharge date, as well as discharge to a new care home, either on a temporarily or permanent basis was also documented [19].

### Categorisation of variables

Dichotomisation was applied for AF, congestive heart failure, hypertension and diabetes, type of stroke, and in-patient infections and mortality according to the presence or absence of any history of the condition. Moderately-severe to severe disability at discharge was defined as an mRS score ≥ 4. Moderately-severe to severe stroke on arrival and at 24-h was defined as an NIHSS score ≥ 16. Prolonged LOS on HASU was defined as those who stayed longer than 2 weeks. Severity of LOC scale during the first 7-days of initial admission was dichotomised into two groups: group 1 with a score of 0 (alert keenly responsive), and group 2 with a score of ≥ 1 (ranging from not alert but arousable by minor stimulation to respond only with reflex motor or autonomic effects/totally unresponsive).

### Statistical analysis

Chi-squared tests were used to assess the proportions of individuals with adverse consequences in relation to different study groups (with or without immediate TRC), and Kruskal–Wallis tests were used to assess group differences in LOS on HASUs and changes in stroke severity. Multivariable logistic regression was conducted to estimate the risk of severe stroke at 24-h, prolonged LOS on HASU, in-patient mortality, UTI and pneumonia within 7-days of admission, severity in LOC scale in the first 7-days, disability at discharge and palliative care by discharge date (dependent variables) from patients with immediate TRC using patients without immediate TRC as the reference group (independent variable). The results are presented as three models: model 1, unadjusted; model 2, adjusted for age, sex and co-morbidities (AF, congestive heart failure, hypertension, diabetes and previous stroke), and model 3, as in model 2 plus time from onset to thrombolysis and NIHSS on arrival, and expressed as odds ratios (OR) and 95% confidence intervals (CI). Analyses were performed using IBM SPSS Statistics for Windows, V.25.0 (IBM Corp., Armonk, NY, USA). The null hypothesis was rejected when *p* < 0.05.

## Results

**Fig. 1:**
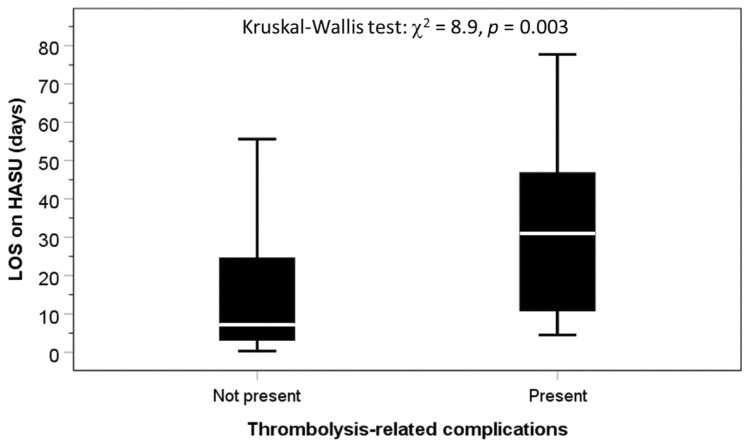
Comparison of length of stay on HASU between patients without and with immediate thrombolysis-related complications

Data from 235 men and 216 women aged 75.3 years (± 13.2) were analysed. Twenty-nine patients (6.4%) developed immediate TRC, mostly symptomatic intracranial haemorrhage (n = 18) (Table 1). The median age (IQR) for patients with immediate TRC was 83 years (75–87.5) and for those without was 77 years (67–85) (Kruskal–Wallis test: χ^2^ = 5.8, *p* = 0.016). The proportions of men (51.2%) and women (48.8%) were similar between groups, while there was a male predominance in the group with immediate TRC (65.5% men: 34.5% women). The rates of AF among patients with immediate TRC were higher (37.9% vs 18.5%, χ^2^ = 6.5, *p* = 0.011), but no significant group differences in the use of anticoagulation therapy for AF: 36.4% vs 27.3%, χ^2^ = 3.3, *p* = 0.192. There were no differences in the rates of other co-existing morbidities including congestive heart failure, hypertension, diabetes and previous stroke, or the rates of patients with moderately-severe to severe NIHSS scores (≥ 16). The proportions of individuals with TRC were higher for a number of adverse consequences in hospital. These included moderately-severe to severe stroke 24-h after thrombolysis, LOC scores ≥ 1 in the first 7-days, and pneumonia within 7-days of admission. There were also higher proportions of HASU stay for ≥ 2 weeks, mortality in hospital and moderately-severe to severe disability (mRS score ≥ 4) on discharge, as well as requirement for palliative care by the date of discharge in this group (Table 2). Compared to patients without TRC, the LOS on HASU was longer by about 6 days for those who had the immediate TRC; Kruskal–Wallis test: χ^2^ = 8.9, *p* = 0.003 (Fig. 1).

**Table 1:** Frequency of immediate thrombolysis-related complications amongst 451 patients undergoing thrombolysis for ischaemic stroke

	n	%
Symptomatic intracranial haemorrhage	18	4.0
Orolingual angioedema	2*	0.4
Extracranial bleed	2	0.4
Abdominal pain	1	0.2
Anaphylaxis	1	0.2
Bleeding gums	1	0.2
Epistaxis	2	0.4
Gastrointestinal bleed	1	0.2
Intracerebral bleed	1	0.2
Asymptomatic haemorrhage	1	0.2
All patients with complications	29	6.4

*One patient with both intra-cranial haemorrhage and orolingual angioedema

**Table 2:** Proportions of adverse outcomes in 451 patients without (n = 422) and with (n = 29) post-thrombolysis complications

	Immediate thrombolysis-related complications		Group difference	
	Not present (%)	Present (%)	χ^2^	*p*
Co-existing morbidities				
Atrial fibrillation	18.5	37.9	6.5	0.011
Congestive heart failure	4.7	6.9	0.3	0.602
Hypertension	55.2	55.2	0	0.997
Diabetes mellitus	15.2	24.1	1.6	0.199
Previous stroke	20.9	24.1	0.2	0.675
NIHSS score ≥ 16 on arrival	14.0	10.3	0.3	0.582
Thrombolysis-related adverse consequences in hospital				
NIHSS score ≥ 16 at 24 h after thrombolysis	5.7	20.7	9.8	0.002
Worst LOC in the first seven days score ≥ 1	25.0	60.7	16.8	< 0.001
UTI within 7 days of admission	5.8	10.7	1.1	0.288
Pneumonia within 7 days of admission	11.0	35.7	14.5	< 0.001
UTI and/or pneumonia within 7 days of admission	13.5	39.3	13.5	< 0.001
LOS in HASU ≥ 2 weeks	34.7	66.7	6.4	0.012
Mortality in hospital	13.0	41.4	17.2	< 0.001
Risk of malnutrition	0.5	0	0.1	0.708
mRS score ≥ 4 on discharge	26.8	65.5	19.7	< 0.001
Level of care support planned at discharge				
Activities of daily living support required by patients	17.6	20.0	0.1	0.815
Joint care planning between health and social care for post-discharge management	24.6	20.7	0.2	0.631
New care home (permanent and temporary)	3.8	6.9	0.7	0.409
New care home (permanent)	2.6	6.9	1.7	0.182
Palliative care by discharge date	5.1	24.1	16.1	< 0.001

*NIHSS* National Institutes of Health for Stroke Scale, *LOC* level of consciousness, *UTI* urinary tract infection, *LOS* length of stay, *HASU* hyperacute stroke unit, *mRS* modified Rankin Scale

**Fig. 2:**
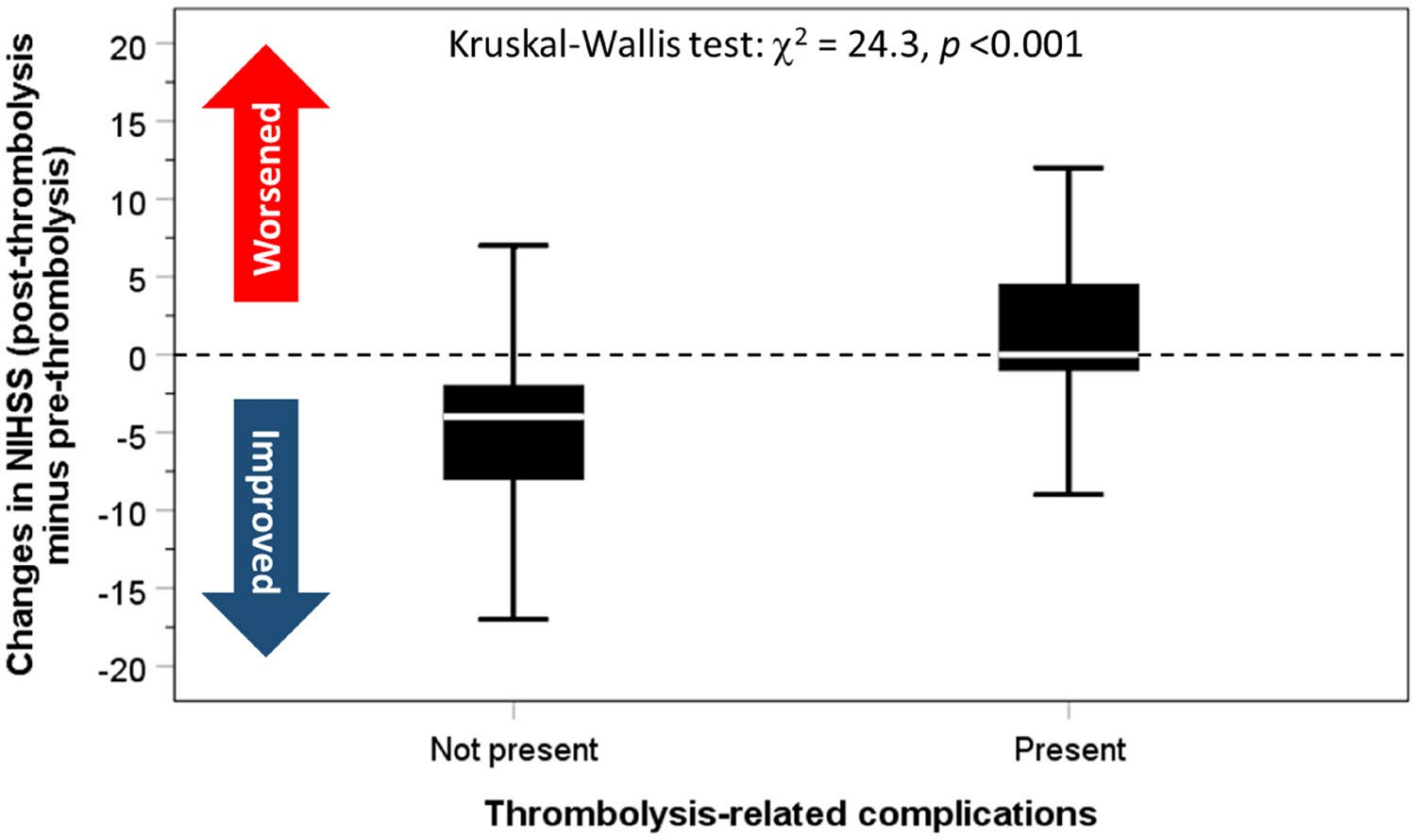
Comparison of changes in NIHSS: post-thrombolysis (at 24-h) minus pre-thrombolysis (on arrival) between patients without and with immediate thrombolysis-related complications

The median (IQR) NIHSS score on arrival was 12 (7–18) and at 24-h post-thrombolysis was 6 (2–12) for those without immediate TRC; corresponding values for those with immediate TRC were 12 (6–18) and 14 (6–20). The change in NIHSS score at 24-h after thrombolysis minus NIHSS score on arrival were − 4 (− 8 to − 2) for patients without immediate TRC and 0 (− 1 to 6) for those with immediate TRC was significant: a difference of − 4 (− 8 to − 1); Kruskal–Wallis test: χ^2^ = 24.3, *p* < 0.001 (Fig. 2).

**Fig. 3:**
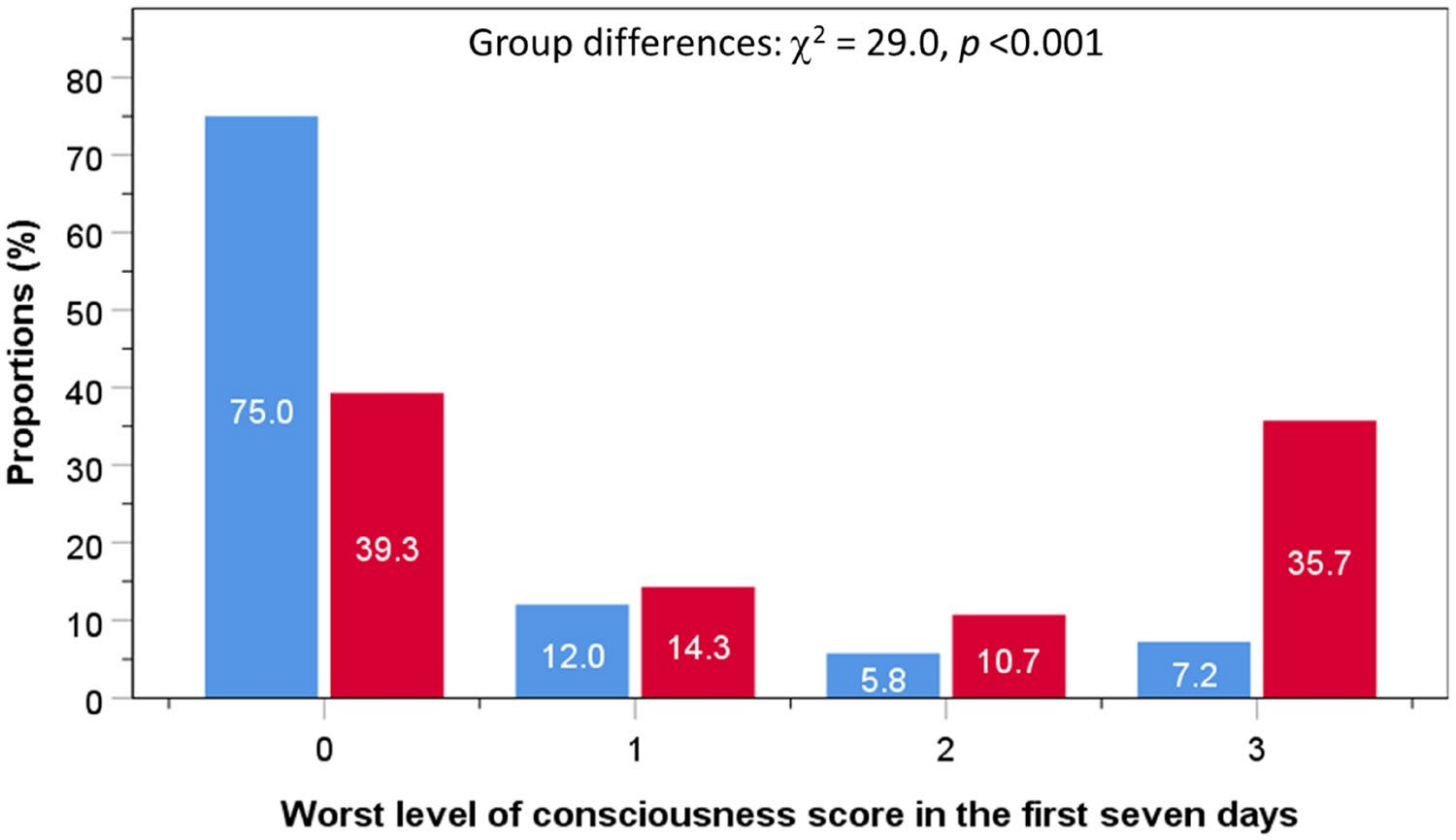
Proportions of patients in different categories of worst level of consciousness score in the first 7-days following initial admission for stroke according to status of thrombolysis-related complications: blue bars indicate no complications, red bars indicate presence of complications

The proportions of patients with a LOC score in first 7-days of 0 (alert) were higher in patients without immediate TRC (75%) compared to those with immediate TRC (39.3%). Conversely, the proportions of patient with immediate TRC rose progressively with increasing LOC scores, peaking at 35.7% for a score of 3 (respond only with reflex motor or autonomic effects/totally unresponsive), compared to 7.2% in those without immediate TRC (Fig. 3).

Compared with patients without immediate TRC (reference group), individuals with immediate TRC had greater adjusted risks of: moderately-severe or severe stroke (NIHSS score ≥ 16) at 24-h (5.7% vs 24.7%, OR 3.9, 95% CI 1.4–11.1); LOC score ≥ 1 in the first 7-days (29.0 vs 60.7, OR 4.6, 95% CI 2.1–10.2); urinary tract infection or pneumonia within 7-days of admission (13.5% vs 39.3%, OR 3.2, 95% CI 1.3–7.7); LOS on HASU ≥ 2 weeks (34.7% vs 66.7%, OR 5.2, 95% CI 1.5–18.4); mortality (13.0% vs 41.4%, OR 3.7, 95% CI 1.6–8.4); moderately-severe or severe disability (mRS score ≥ 4) at discharge (26.8% vs 65.5%, OR 4.7, 95% CI 2.1–10.9); palliative care requirement by discharge date (5.1% vs 24.1%, OR 5.1, 95% CI 1.7–15.7) (Table 3). The median LOS in HASU was longer (7-days vs 30 days, Kruskal–Wallis test: χ^2^ = 8.9, *p* = 0.003) while stroke severity did not improve (NIHSS score at 24-h post-thrombolysis minus NIHSS score at arrival = − 4 vs 0, χ^2^ = 24.3, *p* < 0.001). Additional adjustment for time from stroke onset to thrombolysis and severity on arrival improved the associations only marginally.

**Table 3:** Logistic regression to assess the risk of adverse consequences from immediate thrombolysis-related complications

	OR	95% CI	*p*
Model 1: unadjusted			
NIHSS score ≥ 16 at 24 h after thrombolysis	4.33	1.61–11.62	0.004
Worst LOC in the first seven days score ≥ 1	4.64	2.10–10.23	< 0.001
Pneumonia within 7 days of admission	4.50	1.95–10.35	< 0.001
UTI or pneumonia within 7 days of admission	4.15	1.84–9.33	< 0.001
LOS in HASU ≥ 2 weeks	3.77	1.26–11.27	0.018
Mortality in hospital	4.71	2.14–10.39	< 0.001
mRS score ≥ 4 on discharge	5.20	2.35–11.51	< 0.001
Palliative care by discharge date	6.00	2.28–15.83	< 0.001
Model 2: adjusted for age, sex, co-morbidities			
NIHSS score ≥ 16 24 h after admission	3.90	1.37–11.07	0.011
Worst LOC in the first seven days score ≥ 1	4.14	1.82–9.40	0.001
Pneumonia within 7 days of admission	3.14	1.29–7.66	0.012
UTI or pneumonia within 7 days of admission	3.15	1.33–7.47	0.009
LOS in HASU ≥ 2 weeks	5.23	1.49–18.39	0.010
Mortality in hospital	3.65	1.59–8.41	0.002
mRS score ≥ 4 at discharge	4.73	2.06–10.87	< 0.001
Palliative care by discharge date	4.16	1.39–12.44	0.011
Model 3: as in model 2 plus time from onset to thrombolysis and NIHSS on arrival			
NIHSS score ≥ 16 24 h after admission	5.55	1.65–18.74	0.006
Worst LOC in the first seven days score ≥ 1	7.44	2.77–19.96	< 0.001
Pneumonia within 7 days of admission	3.83	1.47–10.02	0.006
UTI or pneumonia within 7 days of admission	3.59	1.46–8.87	0.006
LOS in HASU ≥ 2 weeks	8.44	2.11–33.73	0.003
Mortality in hospital	3.67	1.59–8.43	0.002
mRS score ≥ 4 on discharge	5.46	2.11–14.13	< 0.001
Palliative care by discharge date	5.10	1.66–15.70	0.004

*NIHSS* National Institutes of Health for Stroke Scale, *LOC* level of consciousness, *UTI* urinary tract infection, *LOS* length of stay, *HASU* hyperacute stroke unit, *mRS*, modified Rankin Scale

## Discussion

Although immediate TRC are well recognised [20, 21], there are no published data on their subsequent consequences. We observed that one in 15 patients acquired immediate TRC in this study. Such individuals, compared to those thrombolysed patients with no immediate complications, had 4–8 fold risk of adverse consequences including moderately-severe to severe stroke (NIHSS score ≥ 16), worst LOC scores in the first 7-days, pneumonia within 7-days of admission, prolonged LOS, in-patient mortality, as well as moderately-severe to severe disability (mRS score ≥ 4) and requiring palliative care by discharge date. As far as we are aware, there are no published reports on changes to stroke severity, based on NIHSS scores, before and after immediate TRC.

Most studies concerning the management of patients undergoing thrombolysis tend to focus on the overall outcomes in all patients as a whole (with or without immediate TRC). Our study provided further evidence on a particular range of adverse consequences specifically arising from patients who developed immediate complications after thrombolysis, including a change of stroke severity, nosocomial infections, disability and the need for palliative care by discharge date, and in-patient mortality. All these adverse consequences were independent of age, sex and a range of major co-existing morbidities. We have also demonstrated that the stroke severity in patients with immediate TRC did not improve after thrombolysis while the severity in patients who were free of immediate TRC was significantly reduced after treatment. There were no group differences in level of care required on discharge.

Variably reported figures of TRC in various studies are likely to be due to differences in patient characteristics such as age, underlying co-morbidities, disability and stroke severity at presentation [22–24], as well as different management approaches, particularly of symptomatic ICH [25–27]. The observation of older age and male predominance in patients with immediate TRC is consistent with findings from previous studies [28, 29]. Moreover, older adults have increasingly been included for this treatment. Our study showed the median age of patients with TRC (83 years) was six years older than that of patients without immediate TRC (77 years). There are conflicting reports on the benefit of thrombolysis in older patients. In their study including those over 80 years, Mishra et al. [7] observed thrombolysis to be beneficial across all ages. A small study of 38 patients older than 80 showed age does not affect outcome when adjusted for stroke severity, time to thrombolysis, glucose level, and history of coronary heart disease [30]. Meta-analysis also showed age does not affect outcomes when compared with controls [31], but a systematic review by the same group found older age was associated with increased mortality and reduced likelihood of regaining favourable outcomes [32]. These differences may arise from a higher rate of immediate TRC that occur more frequently in older patients, as observed in this study.

In recent years, the rates of thrombolysis amongst patients admitted with AIS were 11.1% in Sweden [33] and 11–12% in England and Wales [8]. These figures are slightly lower than our figure (16.4%). The proportion of immediate TRC of 6.4% observed in our study is comparable to those previously reported [13, 34]. The NHS for England set a long-term thrombolysis target of 20% a year (approximately 14,000 patients/year). Assuming that the immediate TRC rate remains unchanged, the number of thrombolysis-related complications is expected to be nearly double the current number (approximately 14,000 × 6.4% = 900 patients/year).

Life expectancy in the UK, as for other industrialised nations [35, 36], is increasing. At the same time in clinical practice there is no age limit for thrombolysis. As a result, according to our observations and the estimated projections listed above, more stroke survivors will be living with dependency, causing a significant impact on the social and healthcare systems. Our findings support the need for more research; not only to lower the rate of immediate TRC but also to prevent adverse outcomes among those who acquire them, and to lower the number with immediate TRC to improve their clinical consequences. Our observations of symptomatic ICH as the most common complication of thrombolysis is consistent with previous studies [13]. Recent studies have provided insights into the mechanisms of intracerebral bleeding after thrombolysis in acute ischaemic stroke and suggested the use of different biomarkers to predict its occurrence [22–24, 37, 38]. Factors such as pre-treatment normoglycaemia, female gender and lower stroke severity are favourable predictors of clinical outcomes after thrombolysis [28, 39], while higher rates of post-thrombolysis ICH increase with hypertension [28, 29, 40, 41], diabetes [28, 41], older age [28, 29], severe stroke [28, 40, 42], adverse fibrinolytic profile at admission [43] and certain biomarkers such raised plasma cellular-fibronectin concentrations [44]. The risk of symptomatic ICH following thrombolysis is associated with pre-treatment antiplatelet agents in some studies [28, 29], but equally this was not demonstrated in others [28, 42]. In this study, the prevalence of AF was higher in the group with immediate TRC than those without (37.9% vs 18.5%, χ^2^ = 6.5, *p* = 0.011). We found that the proportions immediate TRC did not differ significantly between patients who had antiplatelet treatment (10.6%) and those who did not have the treatment (13.9%) prior to thrombolysis (*p* = 0.857). In addition the rate of anticoagulation therapy for their AF prior to thrombolysis was slightly higher, but did not achieve significance and this may be due to the small sample size or selection bias. Therefore these observations cannot be interpreted with confidence. In current practice, the dose of rtPA is based on the patient’s body weight. A more individualised rtPA dose, adjusted according to existing risk factors such as age and premorbid treatment, may reduce bleeding such as ICH.

There are a number of published guidelines for the management of thrombolysis-related haemorrhage. The aim is to reverse fibrinolysis with agents such as fresh frozen plasma, anti-platelet effect with platelet transfusion, and coagulopathy with agents including cryoprecipate [45, 46]. The less common orolingual angioedema is treated with antihistamines and methylprednisolone if required, and further escalation to adrenaline (epinephrine). Where angioedema fails to respond to drug therapy, intubation or tracheostomy may be necessary [47]. As far as we are aware, guidelines for management specifically for patients who acquired immediate TRC after hospital discharge are not available.

### Strengths and limitations

The strength of the present study lies in its large cohort of patients derived from one of the largest NHS regions in the UK and who have similar characteristics to the rest of the UK [8]. Data were collected in accordance with the national SSNAP protocol and analysis took a range of confounding factors known to associate with stroke outcomes into account. The definitions of stroke severity (NIHSS) [17] and disability (mRS) [18] were based on validated tools commonly used for assessment of acute stroke. However, due to the relatively low rates, the number of patients receiving thrombolytic treatment thrombolysis was small. Caution should be taken when comparing results from other populations due to different approaches to the management of immediate TRC that may differ in outcomes.

In conclusion, the risk of nosocomial infections, worsening of stroke severity, longer HASU stay, disability and death is increased following immediate TRC. This finding has major implications for those centres seeking to increase their thrombolysis service to acute stroke patients.

## Data Availability

No additional data are available.

## Declarations

### Research involving human and animal participants

This article does not contain any studies with animals performed by any of the authors.

## References

[1] European Stroke Organisation guidelines for stroke management: Update January 2009. Available from: http://www.eso-stroke.org/ eso-stroke/education/education-guidelines.html. Accessed 3 Mar 2014

[2] Intercollegiate Stroke Working Party. National Clinical Guidelines for Stroke. http://www.rcplondon.ac.uk/resources/ stroke-guidelines. Accessed 2 Apr 2012

[3] Demaerschalk BM, Kleindorfer DO, Adeoye OM, Demchuk AM, Fugate JE, Grotta JC (2016). Scientific rationale for the inclusion and exclusion criteria for intravenous alteplase in acute ischemic stroke: a statement for healthcare professionals from the American Heart Association/American Stroke Association. Stroke.

[4] Fang MC, Cutler DM, Rosen AB (2010). Trends in thrombolytic use for ischemic stroke in the United States. J Hosp Med.

[5] Schwamm LH, Ali SF, Reeves MJ, Smith EE, Saver JL, Messe S (2013). Temporal trends in patient characteristics and treatment with intravenous thrombolysis among acute ischemic stroke patients at get with the guidelines-stroke hospitals. Circ Cardiovasc Qual Outcomes.

[6] Ford GA, Ahmed N, Azevedo E, Grond M, Larrue V, Lindsberg PJ (2010). Intravenous alteplase for stroke in those older than 80 years old. Stroke.

[7] Mishra NK, Ahmed N, Andersen G, Egido JA, Lindsberg PJ, Ringleb PA (2010). Thrombolysis in very elderly people: controlled comparison of SITS international stroke thrombolysis registry and virtual international stroke trials archive. BMJ.

[8] Sentinel Stroke National Audit Programme. Springboard for Progress: The Seventh SSNAP Annual Report. https://www.strokeaudit.org/Documents/National/Clinical/Apr2019Mar2020/Apr2019Mar2020-AnnualReport.aspx

[9] Rodriguez-Castro E, Lopez-Dequit I, Santamaria-Cadavid M, Arias-Rivas S, Rodriguez-Yanez M, Pumar JM (2018). Trends in stroke outcomes in the last ten years in a European tertiary hospital. BMC Neurol.

[10] Darehed D, Blom M, Glader EL, Niklasson J, Norrving B, Eriksson M (2020). In-hospital delays in stroke thrombolysis: every minute counts. Stroke.

[11] Wardlaw JM, Murray V, Berge E, Del Zoppo G, Sandercock P, Lindley RL (2012). Recombinant tissue plasminogen activator for acute ischaemic stroke: an updated systematic review and meta-analysis. Lancet.

[12] Bustamante A, Garcia-Berrocoso T, Rodriguez N, Llombart V, Ribo M, Molina C (2016). Ischemic stroke outcome: a review of the influence of post-stroke complications within the different scenarios of stroke care. Eur J Intern Med.

[13] Bray BD, Campbell J, Hoffman A, Tyrrell PJ, Wolfe CD, Rudd AG (2013). Stroke thrombolysis in England: an age stratified analysis of practice and outcome. Age Ageing.

[14] Royal College of Physicians. Clinical effectiveness and evaluation unit on behalf of the intercollegiate stroke working party. SSNAP January–March 2016. Public Report. https://www.strokeaudit.org/Documents/National/AcuteOrg/2016/2016-AOANationalReport.aspx. Accessed 15 Jan 2021

[15] Han TS, Fry CH, Fluck D, Affley B, Gulli G, Barrett C (2018). Anticoagulation therapy in patients with stroke and atrial fibrillation: a registry-based study of acute stroke care in Surrey, UK. BMJ Open.

[16] Han TS, Gulli G, Affley B, Fluck D, Fry CH, Barrett C (2019). New evidence-based A1, A2, A3 alarm time zones for transferring thrombolysed patients to hyper-acute stroke units: faster is better. Neurol Sci.

[17] Brott T, Adams HP, Olinger CP, Marler JR, Barsan WG, Biller J (1989). Measurements of acute cerebral infarction: a clinical examination scale. Stroke.

[18] van Swieten JC, Koudstaal PJ, Visser MC, Schouten HJ, Van Gijn J (1988). Interobserver agreement for the assessment of handicap in stroke patients. Stroke.

[19] Wang YX, Li YQ, Chen Y, Zhang CH, Dong Z, Wang Z (2018). Analysis of related factors of orolingual angioedema after rt-PA intravenous thrombolytic therapy. Eur Rev Med Pharmacol Sci.

[20] Han TS, Fry CH, Gulli G, Affley B, Robin J, Irvin-Sellers M (2020). Prestroke disability predicts adverse poststroke outcome: a registry-based prospective cohort study of acute stroke. Stroke.

[21] Nesselroth D, Gilad R, Namneh M, Avishay S, Eilam A (2018). Estimation of seizures prevalence in ischemic strokes after thrombolytic therapy. Seizure.

[22] Mazya M, Egido JA, Ford GA, Lees KR, Mikulik R, Toni D (2012). Predicting the risk of symptomatic intracerebral hemorrhage in ischemic stroke treated with intravenous alteplase: safe implementation of treatments in stroke (SITS) symptomatic intracerebral hemorrhage risk score. Stroke.

[23] Menon BK, Saver JL, Prabhakaran S, Reeves M, Liang L, Olson DM (2012). Risk score for intracranial hemorrhage in patients with acute ischemic stroke treated with intravenous tissue-type plasminogen activator. Stroke.

[24] Saposnik G, Fang J, Kapral MK, Tu JV, Mamdani M, Austin P (2012). The iScore predicts effectiveness of thrombolytic therapy for acute ischemic stroke. Stroke.

[25] Goldstein JN, Marrero M, Masrur S, Pervez M, Barrocas AM, Abdullah A (2010). Management of thrombolysis-associated symptomatic intracerebral hemorrhage. Arch Neurol.

[26] French KF, White J, Hoesch RE (2012). Treatment of intracerebral hemorrhage with tranexamic acid after thrombolysis with tissue plasminogen activator. Neurocrit Care.

[27] Yaghi S, Eisenberger A, Willey JZ (2014). Symptomatic intracerebral hemorrhage in acute ischemic stroke after thrombolysis with intravenous recombinant tissue plasminogen activator: a review of natural history and treatment. JAMA Neurol.

[28] Tanne D, Kasner SE, Demchuk AM, Koren-Morag N, Hanson S, Grond M (2002). Markers of increased risk of intracerebral hemorrhage after intravenous recombinant tissue plasminogen activator therapy for acute ischemic stroke in clinical practice: the multicenter rt-PA stroke survey. Circulation.

[29] Larrue V, von Kummer RR, Müller A, Bluhmki E (2001). Risk factors for severe hemorrhagic transformation in ischemic stroke patients treated with recombinant tissue plasminogen activator: a secondary analysis of the European-Australasian acute stroke study (ECASS II). Stroke.

[30] Engelter ST, Reichhart M, Sekoranja L, Georgiadis D, Baumann A, Weder B (2005). Thrombolysis in stroke patients aged 80 years and older: Swiss survey of IV thrombolysis. Neurology.

[31] Emberson J, Lees KR, Lyden P, Blackwell L, Albers G, Bluhmki E, Stroke Thrombolysis Trialists’ Collaborative Group (2014). Effect of treatment delay, age, and stroke severity on the effects of intravenous thrombolysis with alteplase for acute ischaemic stroke: a meta-analysis of individual patient data from randomised trials. Lancet.

[32] Engelter ST, Bonati LH, Lyrer PA (2006). Intravenous thrombolysis in stroke patients of ≥80 versus <80 years of age—a systematic review across cohort studies. Age Ageing.

[33] Vestesson E, Bray B, James M, Paley L, Kavanagh M, Tyrrell P (2016). An international comparison of thrombolysis in England and Wales, and Sweden using national registers. Int J Stroke.

[34] El Tawil S, Muir KW (2017). Thrombolysis and thrombectomy for acute ischaemic stroke. Clin Med.

[35] Christensen K, Doblhammer G, Rau R, Vaupel JW (2009). Ageing populations: the challenges ahead. Lancet.

[36] Kontis V, Bennett JE, Mathers CD, Li G, Foreman K, Ezzati M (2017). Future life expectancy in 35 industrialised countries: projections with a Bayesian model ensemble. Lancet.

[37] Derex L, Nighoghossian N (2008). Intracerebral haemorrhage after thrombolysis for acute ischaemic stroke: an update. J Neurol Neurosurg Psychiatry.

[38] Karaszewski B, Houlden H, Smith EE, Markus HS, Charidimou A, Levi C (2015). What causes intracerebral bleeding after thrombolysis for acute ischaemic stroke? Recent insights into mechanisms and potential biomarkers. J Neurol Neurosurg Psychiatry.

[39] Demchuk AM, Tanne D, Hill MD, Kasner SE, Hanson S, Grond M (2001). Predictors of good outcome after intravenous tPA for acute ischemic stroke. Neurology.

[40] Gilligan AK, Markus R, Read S, Srikanth V, Hirano T, Fitt G (2002). Baseline blood pressure but not early computed tomography changes predicts major hemorrhage after streptokinase in acute ischemic stroke. Stroke.

[41] Derex L, Hermier M, Adeleine P, Pialat JB, Wiart M, Berthezene Y (2005). Clinical and imaging predictors of intracerebral haemorrhage in stroke patients treated with intravenous tissue plasminogen activator. J Neurol Neurosurg Psychiatry.

[42] Schmülling S, Rudolf J, Strotmann-Tack T, Grond M, Schneweis S, Sobesky J (2003). Acetylsalicylic acid pretreatment, concomitant heparin therapy and the risk of early intracranial hemorrhage following systemic thrombolysis for acute ischemic stroke. Cerebrovasc Dis.

[43] Ribo M, Montaner J, Molina CA, Arenillas JF, Santamarina E, Quintana M (2004). Admission fibrinolytic profile is associated with symptomatic hemorrhagic transformation in stroke patients treated with tissue plasminogen activator. Stroke.

[44] Castellanos M, Leira R, Serena J, Blanco M, Pedraza S, Castillo J (2004). Plasma cellular-fibronectin concentration predicts hemorrhagic transformation after thrombolytic therapy in acute ischemic stroke. Stroke.

[45] Hemphill JC, Greenberg SM, Anderson CS, Becker K, Bendok BR, On behalf of the American Heart Association Stroke Council; Council on Cardiovascular and Stroke Nursing; Council on Clinical Cardiology (2015). Guidelines for the management of spontaneous intracerebral hemorrhage: a guideline for healthcare professionals from the American Heart Association/American Stroke Association. Stroke.

[46] Yaghi S, Willey JZ, Cucchiara B, Goldstein JN, Gonzales NR, Khatri P (2017). Treatment and outcome of hemorrhagic transformation after intravenous alteplase in acute ischemic stroke: a scientific statement for healthcare professionals from the American Heart Association/American Stroke Association. Stroke.

[47] Jauch EC, Saver JL, Adams HP, Bruno A, Connors JJ, Demaerschalk BM (2013). Guidelines for the early management of patients with acute ischemic stroke: a guideline for healthcare professionals from the American Heart Association/American Stroke Association. Stroke.

